# Mortality, migration and epidemiological change in English cities, 1600-1870

**DOI:** 10.1016/j.ijpp.2021.05.009

**Published:** 2021-06-17

**Authors:** Romola J. Davenport

**Affiliations:** 1 Cambridge Group for the History of Population and Social Structure, Department of Geography, University of Cambridge, CB2 3EN, Cambridge, U.K.

**Keywords:** Industrial Revolution, infant mortality, migration, early childhood mortality, life expectancy, urbanisation

## Abstract

**Objective:**

This study tests the argument that industrialisation was accompanied by a dramatic worsening of urban health in England.

**Materials:**

Family reconstitutions derived from baptism, marriage and burial records for the period before 1837, and from civil registration of deaths and census populations between 1837 and 1900.

**Methods:**

Age-specific mortality rates are used as indicators of population health.

**Results:**

The available evidence indicates a decline in urban mortality in the period c.1750-1820, especially amongst infants and (probably) rural-urban migrants. Mortality at ages 1-4 years demonstrated a more complex pattern, falling between 1750 and 1830 before rising abruptly in the mid-nineteenth century.

**Conclusions:**

These patterns are better explained by changes in breastfeeding practices and the prevalence or virulence of particular pathogens than by changes in sanitary conditions or poverty. Mortality patterns amongst young adult migrants were affected by a shift from acute to chronic infectious diseases over the period.

**Significance:**

Pathogen evolution, infant care and migration exerted major influences on mortality trends and should be given greater attention in studies of the health impacts of British industrialisation.

**Limitations:**

Evidence of urban mortality rates is very limited before 1837 and may not be fully representative of industrialising populations. Mortality also provides only a partial picture of the health of urban populations and may be distorted by migration patterns.

**Further research:**

There is enormous scope for collaboration between archaeologists and historians to investigate the health of industrial populations, through the triangulation and contextualisation of diverse sources of evidence.

## Introduction

As for unhealthiness, it may well be supposed, that although seasoned Bodies may, and do live near as long in *London,* as elsewhere, yet new-comers, and Children do not: for the *Smoaks, Stinks,* and close *Air,* are less healthful than that of the Country; otherwise why do sickly Persons remove into the Country-*Air?*

Graunt, (1899) [1676], p. 63.

The rapid urbanisation that accompanied the Industrial Revolution in Britain is often argued to have been accompanied by a dramatic worsening of urban conditions, as industrial workers crowded into rapidly built and poorly governed manufacturing and industrial towns (Daunton, 2001; Lewis, 2002; Szreter and Mooney, 1998; Wohl, 1983). However demographic evidence suggests that death rates were much higher in towns in the seventeenth and eighteenth centuries than in the nineteenth century, and that the Industrial Revolution was accompanied by profound improvements in the survival of urban residents, especially infants and rural migrants. This article draws together evidence for long-run trends in mortality rates in English cities to assess whether the Industrial Revolution was associated with marked increases in mortality levels *within* urban populations. Particular emphasis is placed on the effects of migration (rural-urban and urban-rural), and on pathogen evolution, areas where collaborations between archaeologists and historians have exceptional potential. The article describes the period c.1600-1900, but does not discuss the causes of mortality declines after 1870.

**Figure 1:**
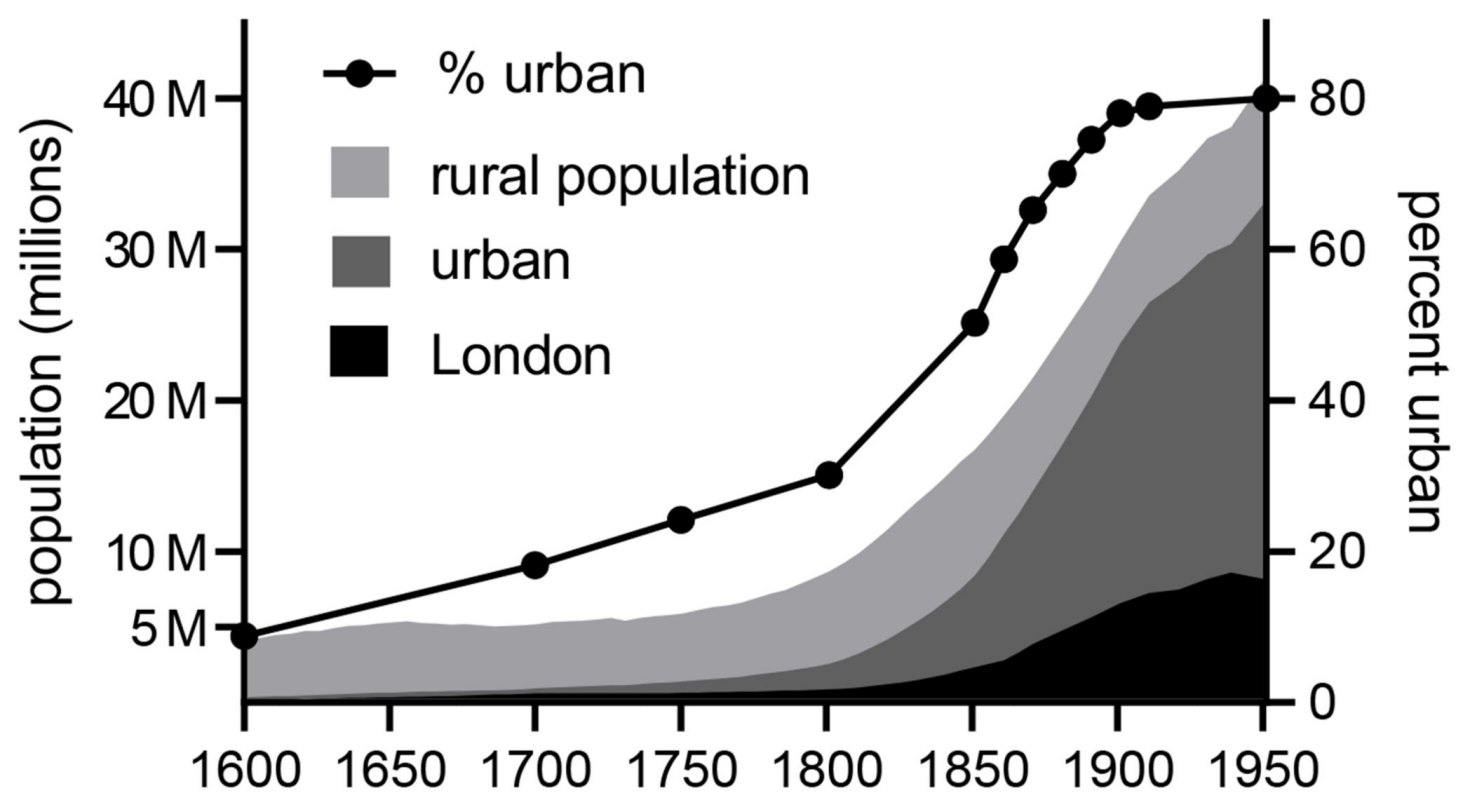
Urbanisation and population growth in England, 1600-1950. Urban figures refer to the population living in settlements of 2,500 or more inhabitants. Estimates for London after 1800 refer to the Metropolis (1801-31), the Registrar-General’s limits (1841-61), and Greater London (18711951). Sources: Bennett, (2012); *Census of Great Britain, 1851* (Vol. I BPP 1852-53 LXXXV (1631)), pp. xxxviii-xxxix; *Census of England and Wales, 1911* (BPP 1912-13 CXI [Cd.6258]), p. xxiv; *Census of England and Wales, 1931,* (BPP 1937) p. 23; Harding (1990); Office for National Statistics (2019); Schwarz (2000); Vries (1984), p. 64; Wrigley et al. (1997), p. 614.

Britain was the first society to become decisively urban. At the time of the first census in 1801 30 % of the English population was already living in urban centres (with populations of 2,500 or more inhabitants, the definition used throughout this paper) (Figure 1). By 1851 over half of the population was urban, peaking at 80 % by 1891. This rapid urbanisation was accompanied from the mid-eighteenth century by unprecedented population growth. The English population doubled between 1740 and 1821, from 5.7 to 11.5 million, and had reached 30.5 million by 1901. The great bulk of this growth was concentrated in towns and cities.

**Figure 2:**
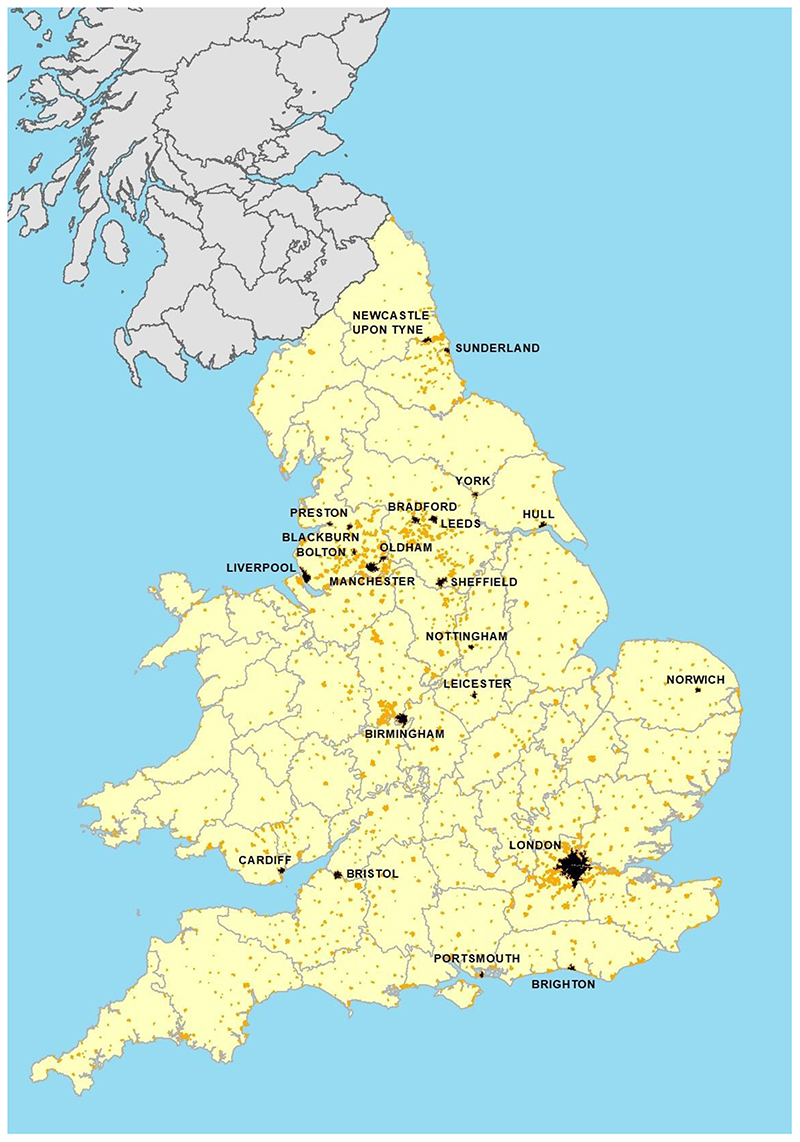
English and Welsh cities with a population of 100,000 or more in 1901. Salford is omitted but is contiguous with Manchester. Black shapes represent the built-up area of each city c.1890, orange shapes the built-up areas of smaller urban centres.

This urban growth was very unevenly distributed. Before 1750 London accounted for the majority of the urban population in England (around two-thirds of the urban population in 1700, and 11 % of the English population as a whole) (Langton, 2000; Wrigley, 1985). By 1700 London had become the largest city in Europe. However London’s growth slowed in the eighteenth century, and the metropolitan population simply kept pace with the growth of the national population, remaining at 10 % of the English population in 1801. A similar pattern in evident amongst most of the major medieval towns, including Bristol, Norwich, and York (Galley, 1998; Wrigley, 1985). In contrast, much more rapid urban growth occurred in smaller industrial and manufacturing towns, located largely on the coalfields (as well as certain ports and leisure towns) (Figure 2). Liverpool grew from around 5,000 in 1700 to 83,000 in 1801 and 376,000 by 1851, and Manchester from around 8,500 in 1700 to 311,000 by 1851 (Langton, 2000; Wrigley, 1985). These cities never approached London in size, however they accounted for an increasing proportion of the urban population. After 1800 the pace of urbanisation accelerated further, and London’s growth also picked up, such that the capital accounted for 14 % of the English population by 1851, and 22 % by 1901. By dint of its size London remained the largest manufacturing centre in Britain throughout the Industrial Revolution, although its economy was more diverse than most of the cities that were more closely associated with the Industrial Revolution (Schwarz, 1992).

Economic historians generally situate the Industrial Revolution in Britain in the century between 1750 and 1850 (Griffin, 2018). This period encompasses the transition from the early mechanisation of many industrial processes, often powered or assisted by water and located in rural areas, to the application of steam-powered technologies and the shift of machinery and factories to urban areas. This was a period of rapid urbanisation, however the limited demographic evidence that we have indicates that early industrialisation coincided with significant improvements in survival, especially in towns (Buer, 2013; Davenport, 2020a; Landers, 1993; Wrigley et al., 1997). Historical debates about the impact of urbanisation and industrialisation on health have instead largely focussed upon the period c.1830-1870, when urban factory labour became common and when urbanisation rates peaked. There is evidence in this period for some deterioration in survival chances especially for young children, but the causes of these reversals remain contested (Davenport, 2020a; Szreter and Mooney, 1998; Woods, 2000).

‘Pessimists’ see the period c.1830-1870 as marking a nadir in living conditions and public health, despite the evidence for rapid growth in GDP per capita (and slower growth in real wages) in this period (Broadberry et al., 2015; Crafts and Mills, 2017; Feinstein, 1998). Simon Szreter has argued forcefully that economic growth necessarily entails disruption, and that in the English case this led to increasing levels of disease, deprivation and death (Szreter, 1997; Szreter and Woolcock, 2004). He also argued that smaller cities that grew rapidly during the Industrial Revolution lacked the institutions of urban governance that older, longer established cities had developed. The limited extension of voting franchise in the 1830s did not remedy this but produced instead a ‘shopocracy’ with narrow self-interest and laissez-fare attitudes to public health and housing, a toxic situation that was only remedied by further extension of the franchise to working class male voters in the late 1860s (Szreter, 1997; Szreter and Mooney, 1998). Evidence of declining stature in institutional populations in the same period suggests a synchronous deterioration in health, although there is vigorous debate regarding the reliability of these samples (Bodenhorn et al., 2017).

Other demographic historians have argued that what is remarkable about the Industrial Revolution period is that England experienced such unprecedented urbanisation and population growth *without* truly catastrophic consequences. Tony Wrigley noted that population growth rates in excess of 1% per year would have resulted in falling real wages and hunger in any previous period (Wrigley, 2011). Indeed while the early stages of the Industrial Revolution (before the mid-nineteenth century) are generally agreed to have produced relatively little improvement in real wages, the fact that wages kept pace at all with increasing population should be viewed as a major achievement (Crafts and Mills, 2020; Wrigley, 2011). In a similar vein, Bob Woods argued that the stagnation of national life expectancy between c.1820 and 1870 actually implied progressive improvements in urban mortality rates, because an ever-increasing proportion of the national population was living in urban areas and exposed to higher mortality rates (Woods, 1985). If urban mortality rates had remained at their eighteenth century levels then a doubling of the urban share of population (as occurred between 1801 and 1861) would have caused a substantial *fall* in national life expectancy, simply as a consequence of these compositional effects.

In this paper I attempt to place mortality developments during the Industrial Revolution in England in a wide chronological and geographical context. I restrict discussion largely to England, because we lack comparable data for other parts of Britain before the mid-nineteenth century.

## 2 Methods and materials

Long-run comparisons of mortality patterns over the period of the Industrial Revolution in England have been severely hampered by the periodisation of source materials, with a marked change in sources after the inception of civil registration of births and deaths in 1837. In order to calculate death rates we require both the numbers of deaths and the size of the population from which the deaths derive, that is, the ‘population at risk’. However before the nineteenth century there was no routine counting of the population, and no centralised reporting of demographic events. Instead, demographic historians rely for the period 1541-1837 principally on Anglican parish registers of baptisms, burials, and marriages (which were required to be kept by law from 1538). These can be used to reconstitute families and to calculate age-specific mortality and fertility rates, a technique known as family reconstitution (Alter et al., 2020; Wrigley et al., 1997). These painstaking reconstructions have been used in conjunction with back-projection from early censuses by Tony Wrigley and colleagues at the Cambridge Group for the History of Population and Social Structure to generate robust estimates of the size and age structure of the English population between 1541 and the nineteenth century. In most cases the estimates of population and mortality rates so derived dove-tail very closely with census (from 1801) and vital registration data (from 1838) (Wrigley et al., 1997).

While these methods have been applied successfully to estimate demographic rates for England and for rural communities and market towns, they work less well for larger urban populations. Large towns are particularly difficult to reconstruct, because they were characterised by unusual age structures (with ‘bulges’ of young adult migrants), often skewed sex ratios (caused by a preponderance of young female domestic servants), and high rates of migration (rural-urban, urban-rural, and urban-urban), and residential mobility within multi-parish towns. Some studies have used ‘partial reconstitution’ to identify family units in relatively short windows of observation, in order to generate estimates of infant and child mortality (Davenport, 2016; Galley, 1998; Newton, 2011). However it is rarely possible to estimate adult mortality rates, because very few adults remained in a single urban parish from birth until death. The only urban reconstitution to produce robust pre-census estimates of adult mortality and life expectancy is John Landers’ study of London Quakers in the period 1650-1849 (Landers, 1993). Quakers recorded births and deaths (rather than baptisms and burials), as well as age and cause of death (in London), and were assiduous in documenting the demographic experiences of chapter members.

Other sources also give insights into urban demographic patterns. Simple tallies of baptisms and burials have been used to track the development of the so-called ‘urban graveyard’ phenomenon, the urban excess of burials over baptisms that was especially pronounced during the period c.1650-1770. The London bills of mortality reported weekly counts of burials by cause of death from the early seventeenth century, and these survive in an almost unbroken series from 1660 to 1849 (with separate tabulations by age group from 1729) (Smith et al., 2020). Other bills of mortality survive for shorter periods for a number of other towns. Superior to these however are the (rare) burial registers that include age and cause of death, because these allow the flexible cross-tabulation of deaths by age and cause (Davenport et al., 2018). These became more common, although still frustratingly rare, in the period c.1778-1812, and age at death was recorded routinely after the imposition of Rose’s Act in 1812 (Basten, 2006). Unfortunately the increasing detail of many burial registers was accompanied by a marked decline in the comprehensiveness and reliability of Anglican burial and baptism registers after c. 1780, as non-conformist sects proliferated and registration practices (especially the timely baptism of new-born infants) became more lax. Additionally, larger towns resorted increasingly to extra-urban burial grounds (Boulton, 2014). Indeed the period 1750-1850 has been described as an urban ‘Demographic Dark Age’ (Galley, 1998, p. 176).

Partly in response to these problems, the English state introduced compulsory civil registration of births and deaths in 1837 (Woods, 2000). In combination with the availability of census data on the size and structure of local populations, this made it possible to calculate age- and cause-specific mortality rates for the whole population. However at present scholars are not allowed free access to death certificates, and are therefore reliant on the Registrar-General’s published statistics. These aggregated data pose very different problems to researchers compared with the individual-level records available for the period pre-1837, and require a different set of analytical techniques (Williams and Galley, 1995; Woods, 2000). Therefore researchers have tended to focus on either the parish register period or the civil registration period, and it has proven very difficult to knit together estimates that bridge the abrupt switch in sources.

**Figure 6:**
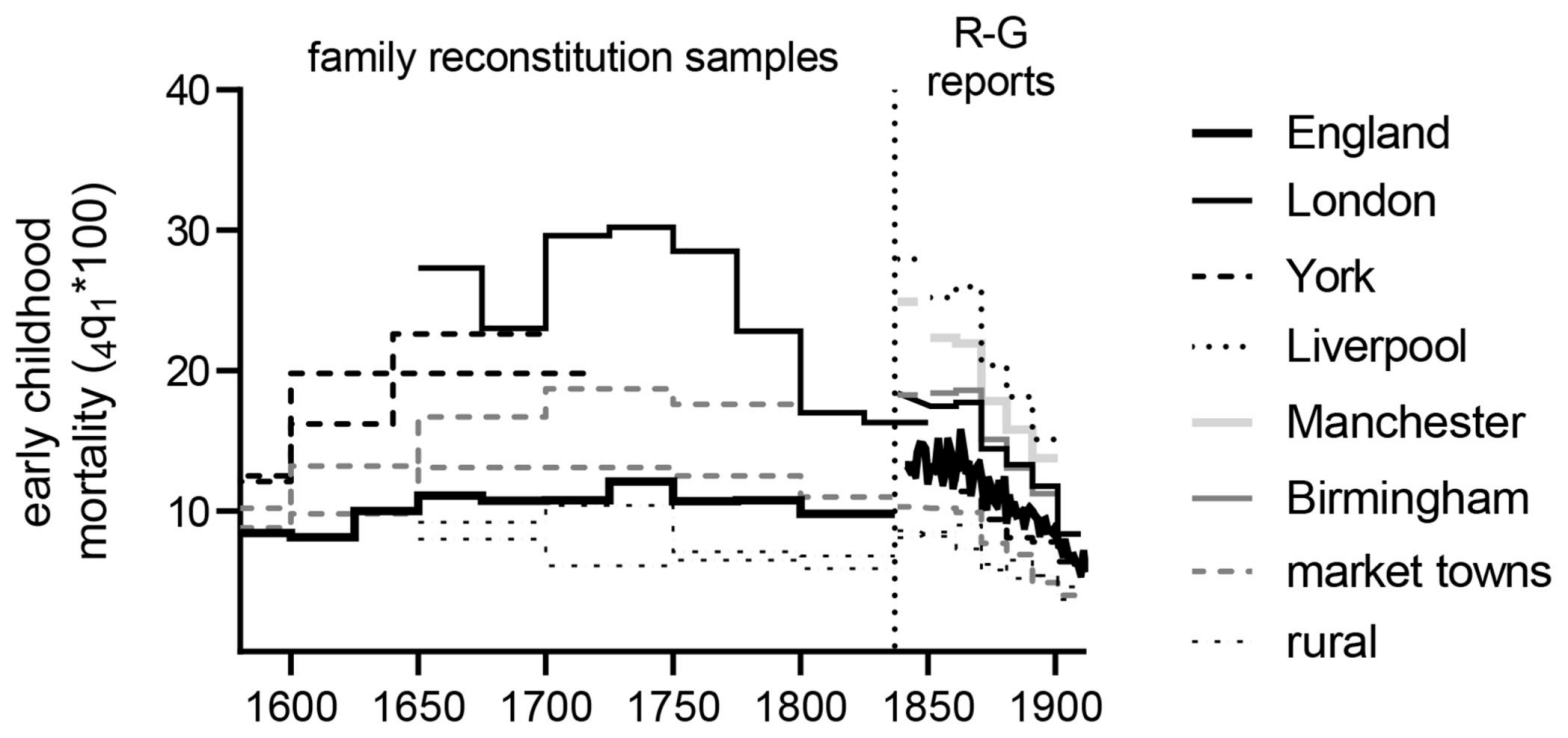
Early childhood mortality (percentage probability of dying between the first and fifth birthdays). See Figure 4 for details and sources. York figures are for two parishes, 1561-1720.

The key demographic measures used in this study are (1) life expectancy at birth (a measure of the average age at death assuming constant age-specific death rates and after adjustments for the age structure of the population) (Preston et al., 2000); (2) infant mortality (deaths in the first year of life per 100 liveborn infants); (3) early childhood mortality (4q1, calculated as the probability of dying between the first and fifth birthdays, per 100 children who survived to age 1); (4) age-specific death rates (as in Figure 6), calculated as annual deaths in each age range, per 100 population in the age range. Note that these demographic rates are calculated here as percentages rather than per 1,000 at risk.

## 3 Results and Discussion

### 3.1 Life expectancy at birth

**Figure 3:**
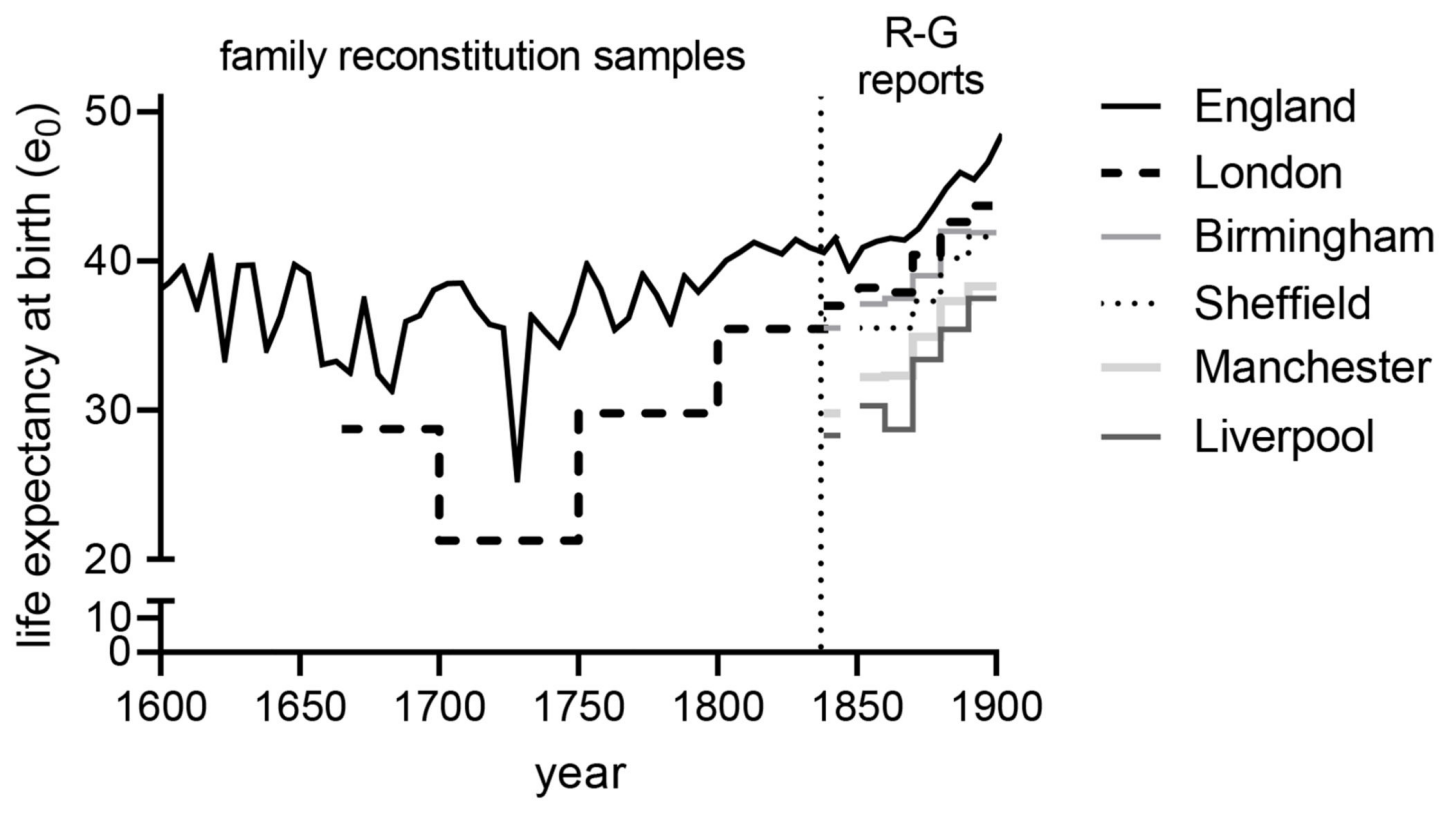
Life expectancy at birth in England and in English cities, 1600-1900. Notes: life expectancy values for England refer to England and Wales after 1840. Data for cities refer to municipal boroughs or urban districts after 1837. Sources: Davenport (2020); Human Mortality Database; Landers (1993), p. 158; Woods (1997); Wrigley et al. (1997), Tab. A9.1.

Figure 3 displays national life expectancy at birth for England in the period 1600-1900, as well as for London Quakers, 1665-1840, and for selected cities in the nineteenth century (1838-1900). At the national level life expectancy was around 37 – 40 years in the early seventeenth century, but worsened in the period c. 1650 – 1740, coinciding with a period of relatively modest fertility and of stagnant population growth. London-born Quakers, a relatively affluent and apparently clean-living group, had estimated life expectancies at birth well below those of the national population, and as low as 21 years in the period 1700 – 1749, a level that implies either very high fertility to balance losses due to mortality, or a population dependent on migration for its perpetuation.

Life expectancy recovered in the second half of the eighteenth century, in both the national and London Quaker populations, and attained new heights in the first decades of the nineteenth century. However national life expectancy stagnated again in the period c.1820 – 70, before improving fairly continuously to the present. Notably, this period of stagnation coincided more or less with the most rapid phase of urbanisation in England, when the urban portion of the population grew from 4.2 to 14.0 million people (Figure 1). Surprisingly however, this mass movement of the population from rural areas with modest mortality rates to towns with much higher mortality rates did not cause a pronounced downturn at the national level (Woods, 1985).

By the mid-nineteenth century, when civil registration of births and deaths permitted more robust estimates of urban life expectancies, life expectancy in London was only four years less than the national average. This is remarkable given the huge size and rapid growth of London, which increased from around 860,000 inhabitants in 1801 to almost two million inhabitants in 1841, and four and a half million by 1901.

London may however be anomalous. Its huge size meant that it comprised a great range of environments. Life expectancy varied markedly between districts, so that London’s high average life expectancy reflected to some extent the contribution of very affluent and salubrious suburbs, as transport improvements facilitated the longstanding historical propensity for residential segregation by socioeconomic status (Woods, 2000, pp. 375-9). In addition, London was the focus of relentless inspection by Parliament, which sat at Westminster (in central London).

Other large cities in England, while much smaller than London, generally had lower (in some cases much lower) life expectancy than the metropolitan population by the mid-nineteenth century (Table 1, Figure 3). Liverpool, England’s second largest city and its second port, with a population of 376,000 in 1851, was infamous for its high mortality rates, and life expectancy was as low as 28 years in the period 1838-44. Manchester, only slightly smaller than Liverpool in size, experienced similarly low levels of life expectancy in the mid-nineteenth century, but also experienced more rapid improvements. Manchester and Liverpool provide some support for the argument that large northern industrial and manufacturing cities experienced much worse conditions, and higher mortality rates, than their slower-growing southern counterparts, most notably Bristol (Szreter and Mooney, 1998). However other large and rapidly growing northern and midlands towns and cities, were less conspicuously lethal (Table 1, Figure 3).

More surprisingly, even the most notorious industrial and manufacturing cities had higher life expectancies than London Quakers a century earlier (Figure 3). And all were capable of natural growth (births exceeded deaths). That is, after several centuries in which many even relatively small cities appear to have been dependent on migration to maintain their populations, large cities had become capable of self-sustaining population growth.

Life expectancy represents the combined effects of mortality at all ages, but not all ages were equally affected by urban conditions. As John Graunt noted with respect to seventeenth century London (in the quote at the beginning of this article), urban conditions exerted their most malign influence on young children, and on recent immigrants. Only roughly half of Quaker children born in London in the seventeenth and early eighteenth centuries survived to age ten. In contrast, married couples (who had married in London, and were long-term residents) could expect to live an average of 26-33 years after their thirtieth birthday, a life expectancy very similar to their peers in more rural communities (Landers, 1993, p. 136,158). Sections 3.2 and 3.3 discuss trends in infant and childhood mortality, before turning in section 3.4 to migrants.

### 3.2 Infant mortality

**Figure 4:**
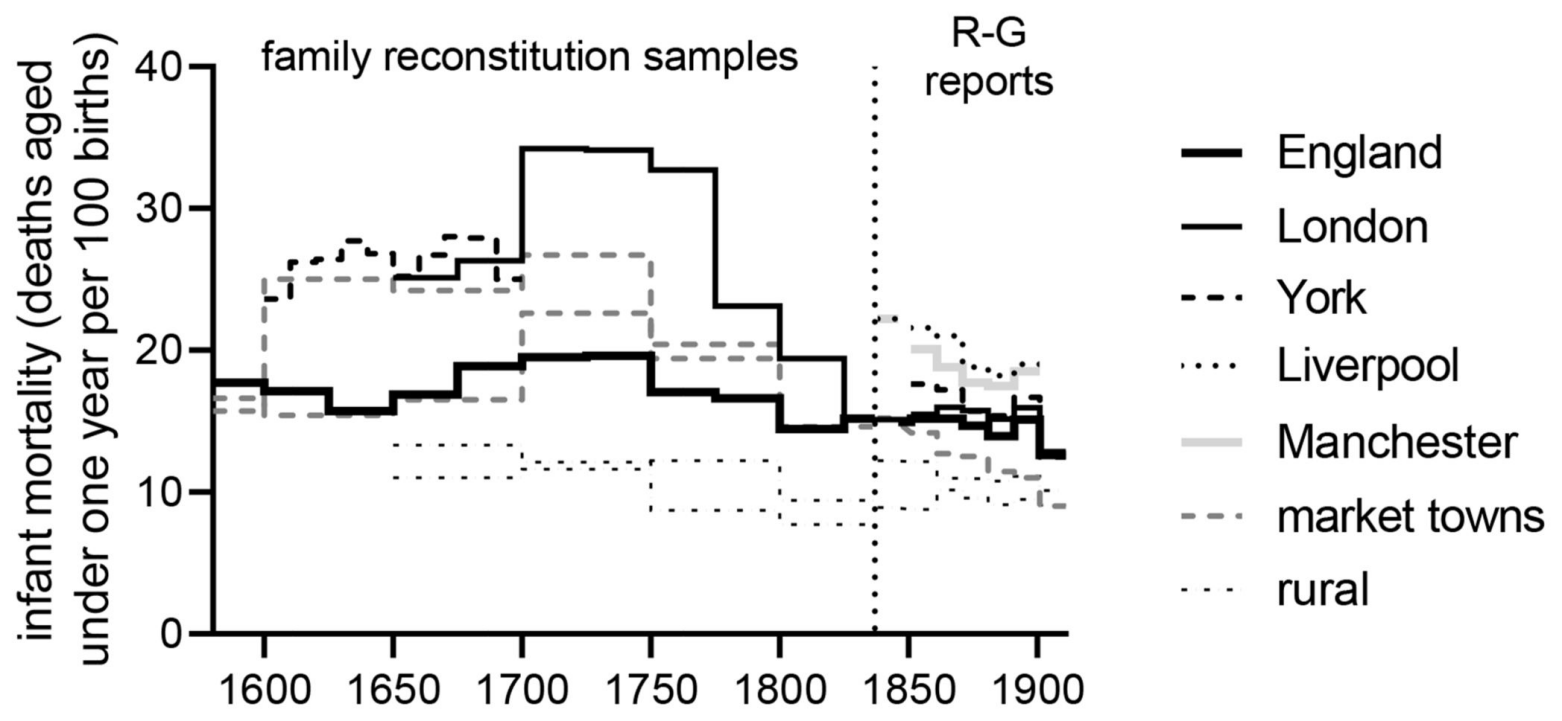
Infant mortality (%) in England and selected English settlements, 1600-1911. Rates for England refer to England and Wales after 1845. Values for London refer to London Quakers before 1850, and to the registration county of London from 1851. York refers to weighted mean of 14 parishes 1601-1700. Market towns refer to Banbury and Gainsborough, rural parishes to Ash (Kent) and Morchard Bishop (Devon). Values refer to those parishes before 1838, and to their corresponding registration districts after that date. Data for other cities refer to municipal boroughs or urban sanitary districts after 1837. Sources: Annual Reports of the Registrar General; Cambridge Group family reconstitutions; Davenport, 2020a; Galley, 1998; Woods, 1997; Wrigley et al., 1997, p. 93.

Estimates of infant mortality (mortality in the first year of life) for England and for sub-populations of a number of English towns and cities are displayed in Figure 4. Infant mortality in the English population (indicated by the heavy black line) broadly tracked life expectancy trends. Nationally, infant mortality rose in the period 1650-1740, to almost 20 % of all live-born infants. Infant survival then improved until about 1820, before stagnating across the rest of the nineteenth century, a phenomenon attributed to high infant mortality in urban areas. This latter pattern was in contrast to life expectancy, which improved after c.1870.

Infant survival in urban centres mirrored national trends in exaggerated form. Infant mortality in London rose from around 25 % of all live births in the mid-seventeenth century to average rates as high as 35 % in the early eighteenth century. York, with a population of only around 12,000 across the seventeenth and early eighteenth century, experienced rates of 24-28 % in the seventeenth century. Even smaller towns of a few thousand inhabitants, such as Banbury in Oxfordshire or Gainsborough in Lincolnshire, experienced infant mortality rates well in excess of the national average, and as high as those recorded in the most lethal industrial cities a century later. For example, Liverpool, notorious for its high mortality in the nineteenth century, recorded average infant mortality rates of 24 % in the 1850s, when its population was at least 300,000, rates comparable with market towns in the early eighteenth century.

The available data indicate that infant mortality fell everywhere in England after around 1750, but fastest in London and in market towns. The greatest improvements occurred in early infancy (the first month of life, or neonatal period), where maternal health is a major factor in infant survival. Neonatal mortality was higher in urban than in rural populations in the two centuries before 1750, however rates subsequently converged, such that by the mid-nineteenth century mortality in the first month of life was very similar across a range of environments (Smith and Oeppen, 2006; Wrigley et al., 1997, pp. 223-76). The large falls in neonatal mortality in towns after 1750 remain poorly understood. Wrigley demonstrated that the decline in neonatal mortality in the national population was associated with an increase in maternal fecundity in the eighteenth century, which he associated with reductions in late foetal mortality (stillbirths) as a result of improvements in maternal health. He attributed these putative improvements to either a reduction in infections during pregnancy (as a result of the endemicisation of many infectious diseases, and their reduction to childhood diseases: Wrigley et al., 1997, p.317-8), or to improvements in diet and nutritional status (Wrigley, 1998). Woods also argued for the importance of reductions in maternal infections, especially smallpox (Woods, 2009), as well as for the contribution of improvements in obstetric practices (Woods and Galley, 2014). Neonatal mortality was very high not only in urban parishes but also in low-lying marshy rural parishes in the seventeenth and early eighteenth centuries. Both urban and low-lying areas were characterised by high levels of infectious diseases, suggesting that the disease environment may have played a larger role than nutrition in these patterns (Smith and Oeppen, 2006).

Other urban-specific factors may also have been at work, most notably breastfeeding practices. The English population as a whole was characterised by relatively high rates of maternal breastfeeding over the sixteenth to early nineteenth centuries, and the average age at weaning is estimated to have been around eighteen months (although the duration of exclusive breastfeeding may have been limited to 4-6 months) (Wrigley et al., 1997). However urban populations were characterised by a much more diverse array of infant feeding practices, including rural wet-nursing, hand-feeding and in-house wet nursing, and by considerable changes in the prevalence of these practices over time.

The practice of sending new-born infants out of urban areas to be nursed in the countryside was common in London until the late eighteenth century. The scale of this export of infants is attested by records of the burials of thousands of private nurse children in rural parishes, and by the marked paucity of young children in more affluent parishes recorded in the quasi-censuses associated with the Marriage Duty Act of 1695 (Clark, 1987; Fildes, 1988; Finlay, 1981, pp. 146-9; Newton, 2011). The practice was most prevalent in wealthy families, but was also evident amongst families of artisans and small traders (Clark, 1987). In the wealthy central London parish of Cheapside 80 % of children aged under 3 years and assumed to be alive were omitted from the 1695 assessment, compared with only 20 % of older children (Newton, 2011). Indirect estimates of child absence suggested that nearly a quarter of all infants were absent at nurse in the large parish of St Martin in the Fields in the mid-eighteenth century, and 40 % of infants in the wealthiest decile of families (Davenport, 2019). The tendency to bury nurse children in their nurse’s rural parish is therefore likely to cause an underestimation of infant mortality rates, and to bias comparisons by social status (whether based on urban parish registers, or on archaeological evidence from urban burials) (e.g. DeWitte et al., 2016). Newton and Finlay considered that the under-recording of deaths of infants at nurse was the main reason why infant mortality appeared to be lower in richer inner London parishes than in the poorer suburban areas (Finlay, 1981, pp. 133-50; Newton, 2011). In St Martin in the Fields, adjustments for the effects of rural wet-nursing revealed a distinct survival disadvantage for wealthier infants across the first year of life, consistent with shorter average birth intervals and lower rates of maternal breastfeeding in wealthier families in the mid-eighteenth century (Davenport, 2019).

Anecdotal and indirect demographic evidence (from birth intervals) indicates that the practice of rural wet-nursing waned in the late eighteenth century, in tandem with a rise in maternal breastfeeding, and also possibly a rise in hand-feeding of infants at home (Davenport, 2019; Fildes, 1986; Landers, 1990). The decline of private wet-nursing coincided in London with the establishment of the London Foundling Hospital and its adoption of the practice of rural wet-nursing for its charges, which may have lowered the social status of rural wet-nursing, as well as making it more difficult to obtain the services of wet-nurses. Rural wet-nursing was also common in Scottish towns, but it remains unclear whether it was widespread in English towns outside London (Marshall, 1988). Exclusive breastfeeding generally postpones conception and lengthens the interval to the next conception through the physiological mechanism of lactational amenorrhea, and therefore comparison of birth intervals can indicate differences in breastfeeding practices, in populations not otherwise practicing contraception. Average birth intervals were shorter in market towns and in wealthier parishes in seventeenth century York (after adjustment for the effects of higher infant mortality in cutting short maternal breastfeeding and hastening conception) (Galley, 1998, pp. 117-22; Wilson, 1986). This suggests high rates of hand-feeding or wet-nursing of urban infants, but we cannot determine whether this occurred within the home parish, or in rural areas.

By the mid-nineteenth century, when we have evidence of infant mortality for a range of towns, English urban centres still demonstrated a marked urban penalty, compared to the national average. However rates in the largest English towns were unexceptional when compared with rates in many smaller European cities (Table 2). Indeed, even in Liverpool, with infant mortality rates of 21 % in the 1860s, rates did not exceed the *national* rate for Germany and were substantially lower than those recorded for Munich and Berlin. Infant diarrhoeal diseases accounted for a significant proportion of the urban penalty for infants in European cities, and these diseases were disproportionately concentrated amongst weaned infants. Many continental populations practiced very limited breastfeeding (Kintner, 1985; Thorvaldsen, 2008; Vögele, 1994), and the comparatively favourable rates of infant mortality in English cities imply relatively high levels of maternal breastfeeding in these populations. The very limited evidence from surveys regarding the prevalence and duration of breastfeeding in selected English cities in the first decade of the twentieth century indicates that breastfeeding was very common at least in the first three months of life, and that there was little apparent difference in rates between industrial or manufacturing towns and other urban centres (Fildes, 1992; Woods, 2000, pp. 285-9). The relatively moderate infant mortality rates in England’s rapidly growing cities is stark testament to the capacity for breastfeeding to buffer infants against even very adverse environments.

Other environmental and domestic factors were also key. Water provision and quality improved markedly in English cities over the period c.1850-1900, in tandem with marked declines in water-borne diseases including cholera, typhoid and dysentery (Davenport et al., 2019; Hamlin, 1988; Hassan, 1985; Hinde and Harris, 2019; Wohl, 1983). Infant diarrhoeal mortality however showed no improvement until after 1900. This suggests that other factors apart from water quality, including dry faecal disposal methods and possibly fly-borne transmission, were important in infant diarrhoeal diseases. More surprisingly, infants seem to have been protected to some extent from diseases with more demonstrable dependence on polluted water, most notably cholera (Davenport et al., 2019), leading some scholars to suggest that (unboiled) water probably played little role in infant diets (Woods, 2000, p. 331).

### 3.3 Early childhood mortality (ages 1-4 years)

**Figure 5:**
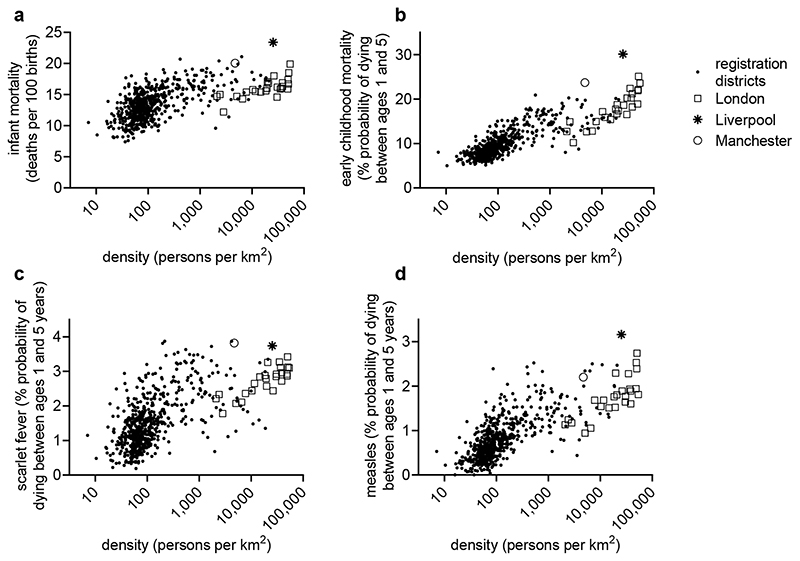
Mortality in registration districts in relation to population density, England and Wales 186-170. Rates are expressed as percentage probability of dying under age 1 year (a), and between exact ages 1 and 5 years from any cause (b), from scarlet fever (c) and from measles (d). ‘London’ refers to registration districts within London registration county, ‘Liverpool’ and ‘Manchester’ to the core registration district of each city. Source: Woods (1997).

Young infants are buffered to some extent against environmental conditions by maternal antibodies acquired in utero, and by the more generalised protective effects of breastmilk (if breastfed). However these effects wane over the first year of life, and therefore early childhood mortality (at ages 1-4 years) generally provides the most sensitive demographic indicator of urban disease environments. Figure 4 shows the relationships of infant and early childhood mortality to population density (measured at the level of the registration district) in mid-nineteenth century England. In infancy the effects of population density appear to level off at fairly modest densities (Figure 5a), and this is also the case for diarrhoeal mortality (Woods, 2000, p. 328). In contrast, the effects of density on mortality in early childhood (ages 1-4 years) were roughly linear, and this was also the case for some of the major childhood diseases, including measles and, to a lesser extent, scarlet fever (Figure 5b-d).

Figure 6 shows long-run trends in early childhood mortality by settlement type. As was the case for infants (Figure 4), there was a rise in mortality across the seventeenth century both nationally and in towns, and some improvement after c.1750. Amongst London Quakers (London prior to 1850), the rise and very marked fall of early childhood mortality was accounted for in large part by trends in smallpox mortality, which accounted for nearly half of all deaths at ages 2-4 years in the eighteenth century (Landers, 1993, 152-6). In the London bills of mortality there was also a marked rise in smallpox as a proportion of all burials across the late seventeenth century. By the mid-eighteenth century, when we have data for more towns, smallpox was the leading cause of death in London and in northern towns, accounting for 6 – 10 % of all burials in London, and fully 20 % in Manchester (Davenport et al., 2018, 2016).

The advent of vaccination led to very rapid falls in smallpox mortality. Amongst London Quakers smallpox and early childhood mortality fell after 1750, suggesting the early adoption of (relatively expensive) inoculation, a forerunner of vaccination. However in most urban centres smallpox mortality fell decisively only after 1800, with the widespread adoption of vaccination (Davenport et al., 2016; Mercer, 2014). These falls coincided with, and probably largely account for, very significant improvements in child survival amongst London Quakers, and in market towns. It is also likely that the rise and fall in smallpox infections between the seventeenth and nineteenth centuries made a significant contribution to infant mortality trends in this period, as a result of smallpox infections in later infancy (as maternally-acquired immunity declined), and possibly via effects of maternal infections on foetal health (Woods, 2009).

The historical evidence for a rise in smallpox mortality in the seventeenth century has been given greater weight by recent ancient DNA (aDNA) evidence. Screening of a large sample of medieval remains indicated that smallpox was present in Europe by at least the 6^th^ century B.C.E.. However none of the strains isolated were closely related to modern strains (Mühlemann et al., 2020). Instead a smallpox strain isolated from a seventeenth century Lithuanian mummy appears to be ancestral to all modern smallpox variants (of both variola major and variola minor) (Duggan et al., 2016). This pattern is consistent with the emergence or introduction of a novel smallpox strain in seventeenth century Europe (Carmichael and Silverstein, 1987). It is also likely that control measures including inoculation and vaccination exerted substantial selective pressures on the subsequent evolution and radiation of modern smallpox strains (Ferrari et al., 2020).

In contrast to trends in infant mortality, falls in early childhood mortality in the late eighteenth and early nineteenth centuries were dramatically reversed in the middle decades of the nineteenth century. Nationally, the time series of early childhood mortality points to an abrupt discontinuity in the 1830s and 1840s (Figure 6). This discontinuity coincided with the introduction of civil registration of births and deaths in 1837, and the shift from estimates based on family reconstitution of a small set of mainly rural and market town communities to rates based on the national population, suggesting that it reflected the absence of large towns from the earlier series. However the discontinuity was confined to the age group 1-4 years, and was evident in rural areas as well as market towns (Davenport, 2020a). After intensive consideration of and adjustment for the impact of these changes in data quality and recording, Wrigley and colleagues concluded that the most likely explanation for the rise in early childhood mortality in this period was not a compositional effect, but some dramatic change in the disease environment (Wrigley et al., 1997, pp. 255-61).

A plausible candidate is scarlet fever. It is widely accepted by demographic historians that scarlet fever underwent autonomous changes in virulence in the nineteenth century, and that a decline in virulence was important in reducing childhood mortality after c. 1870 (Creighton, 1894; Katz and Morens, 1992; Swedlund and Donta, 2002). A survey of demographic evidence for rural populations, towns and cities across Europe and north America in the first half of the nineteenth century indicated that mortality in early childhood rose more or less synchronously across these regions in the fourth and fifth decades of the nineteenth century, in association with the abrupt onset of large epidemics of scarlet fever (Davenport, 2020a). A rise in scarlet fever mortality could explain why early childhood mortality rose in rural as well as urban areas in the period c.1830-70 (although the dependence of scarlet fever mortality on population density (Figure 5c) meant that the absolute impact on urban children was much larger).

In addition, Liverpool and Manchester experienced much higher mortality in both infancy and early childhood than other districts of comparable densities (evident in Figures 4-6). While Manchester and Liverpool exemplified the ‘shock’ cities of the Industrial Revolution, they also suffered specific problems that were not typical of most industrial and manufacturing cities in this period, including very substantial populations of often destitute Irish migrants (Woods, 2000, p.370). It is important to recognise that levels of early childhood mortality in the largest cities in the nineteenth century generally did not exceed those observed in *small* towns in the seventeenth and eighteenth centuries, with the exception of Liverpool and Manchester, where rates may have approached those of London in the earlier period (Figure 6).

### 3.4 Rural-urban migrants

The very high death rates of early modern cities meant that urban populations depended heavily on migration for persistence and growth. This dependence on migrants diminished to some extent as cities became capable of natural growth, in the late eighteenth century. Most migrants were teenagers and single young adults, and these accounted for the bulge of young adults in the age structures of towns and cities (Day, 2020, 2018; Galley, 1995; Souden, 1984).

We have only fragmentary estimates of the migrant composition of urban centres before the 1841 census, the first to collect information on migration. Migrants appear to have comprised a significant majority of the adult population of London as well as smaller towns until at least the mid-nineteenth century (Table 3; Souden, 1984; Wallis, 2014 Table 6.3). By 1851, when the census included a question on birthplace, migrants comprised the majority of adults in all rapidly growing industrial and manufacturing cities, a necessary concomitant of their rapid growth (Table 3). Female migrants also outnumbered males in most English towns and cities, as a consequence of high demand for female domestic servants (although intra-urban sex ratios could vary markedly depending on local economic activities-see the PopulationsPast website, Reid et al., 2018, to explore migration patterns and sex ratios for small areas in the census years 1851-1911). The very high contribution of young adult migrants to urban populations, sex-selective migration, and intra-urban variations in population composition must be borne in mind especially when examining adult skeletons for evidence of the impact of urban conditions in childhood, socioeconomic differences in health and survival, and urban weaning practices.

Migration status was not recorded in birth or death records, and this makes it difficult to compare mortality between migrants and non-migrants directly. Graunt observed of seventeenth-century London that mortality fell heavily on those newly arrived in London, who were not ‘seasoned’ to urban diseases. Rural-urban migrants often lacked prior exposure and immunity to diseases that were common in urban environments, including typhus, typhoid, diarrhoeal and respiratory diseases, to which frequent exposure conferred some immunity on long-term urban residents (McNeill, 1980). However we can only infer the size of these effects indirectly.

Smallpox posed a particular threat to young adult migrants in the eighteenth century, at least in southern England. For the London-born, smallpox was a disease of childhood, however fully 20 % of all London smallpox burials were of young adults in the mid-eighteenth century, and most of these would have been recent immigrants (Davenport et al., 2011; Landers, 1993; Meier, 2009). In contrast, very few adults died of smallpox in the rapidly growing towns of northern England. Fewer than 5 % of smallpox burials were aged 10 years or more in Manchester in the second half of the eighteenth century, implying that most immigrants had already acquired immunity. Recent work on English smallpox patterns in the eighteenth century (before the advent of mass vaccination c.1800) indicates that smallpox was a childhood disease in most of northern Britain, but remained a rarer disease of adults as well as children in southern England outside the largest cities (probably as a consequence of local poor law policies to avert epidemics: Davenport et al., 2018; Razzell, 2003). Therefore migrants from northern Britain enjoyed an immunological advantage over their southern counterparts, with respect to smallpox.

Although young adult rural-urban migrants were at higher risk of death from some urban diseases than their urban-born peers on account of their immunological naiveté, they may have enjoyed health advantages in other respects. Children in rural areas were generally less exposed to infectious diseases during development, and may have been less stunted than their urban counterparts (Jaadla et al., 2020; Kirby, 2013). In addition, labour migrants are often positively selected for health, as well as other characteristics such as education, a phenomenon dubbed the ‘healthy migrant effect’ (e.g. Kennedy et al., 2015). There is scattered evidence for positive selection for rural-urban migrants in the English past. A much higher proportion of both male and female migrants to London could sign their names, compared with the national population (Earle, 1989; Whyte, 2000), and they may also have been taller than their peers (Humphries and Leunig, 2009). We do not know however whether any such health advantages waned with duration of residence in towns, as has been observed in late nineteenth-century France (Kesztenbaum and Rosenthal, 2011).

Health-selective migration may have played a large role in urban mortality patterns in the nineteenth century, especially with respect to tuberculosis. Respiratory tuberculosis was the leading cause of death in young adults in Victorian England, and was held responsible for over 40 % of all deaths at ages 15-34 in the 1850s (Woods, 1997). It was associated with poverty and with poor nutrition, but also with certain environments to which migrants gravitated, including the textile industries, and more generally, crowded and poorly ventilated workspaces and dwellings. These conditions were especially common in towns. However tuberculosis was a chronic infectious disease, in which the progression from clinical symptoms to death extended over months or years. This allowed would-be migrants to adjust their behaviour according to their health status, and therefore any additional risks of tuberculosis associated with urban conditions did not necessarily manifest as higher death rates in towns.

**Figure 7:**
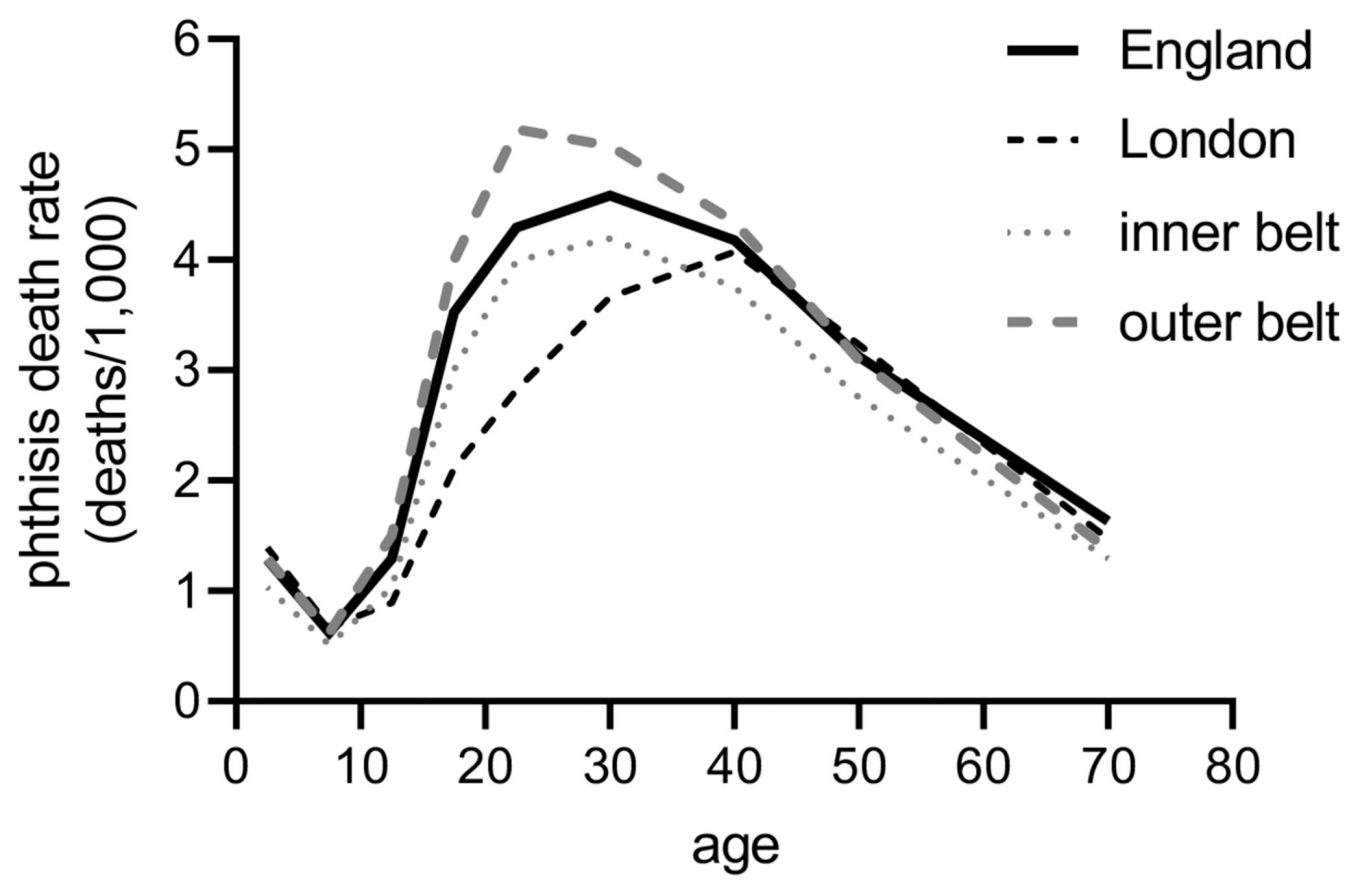
Respiratory tuberculosis (‘phthisis’) mortality rates, females, 1851-60. Age-specific rates are plotted at the midpoint of each age group. Registration districts included in each area are given in the source: Welton (1871), Table V.

Figure 7 plots female respiratory tuberculosis rates in the 1850s, from a study by Thomas Welton (Welton, 1871). The rates for the national population show the typical and highly unusual age pattern of respiratory tuberculosis mortality, which peaked in early adulthood. Also shown are death rates in London, and in districts in two concentric circles around London. Welton drew attention to the surprisingly low rates of respiratory tuberculosis mortality in London, especially in the age groups 15-34 years, when mortality from the disease was highest. Conversely, the mainly rural districts in what Welton termed the ‘outer ring’ (extending to parts of Norfolk, Suffolk, Essex, Cambridgeshire, Northamptonshire, Oxfordshire, Hampshire, Buckinghamshire and Sussex) reported extremely high levels of mortality from respiratory tuberculosis in early adulthood, well in excess of national rates, and peaking at the peak age of migration, in the 20-24 year old age group.

Welton proposed two explanations for this pattern. First, he argued that London’s low death rates were sustained by the constant influx of relatively healthy migrants, a high proportion of whom migrated from districts in the ‘outer ring’. Conversely, the emigration of healthy individuals would have acted to raise mortality in these districts, because the large-scale departure of the well left behind a population enriched with sick young adults (Welton, 1875) (a similar phenomenon can be observed amongst males in civilian populations during periods of mass conscription). More importantly, in Welton’s view, these healthy migrant effects were reinforced by selective patterns of *return* migration. That is, when migrants to the capital developed tuberculosis then they often returned to their home parishes, where they were better able to access financial, medical and informal support (and where they often retained a legal right to support). This type of return migration has been dubbed the ‘salmon bias’ effect (e.g. (Ginsburg et al., 2016; Puschmann et al., 2017; Turra and Elo, 2008). It is very difficult to confirm without well-kept population registers. However several recent studies have provided indirect evidence for the operation of selective return migration in nineteenth-century Britain. Andy Hinde argued that the strong geographical patterning of tuberculosis mortality, and of sex differentials in tuberculosis mortality among young adults, could be explained in terms of sex-selective and health-selective migration, with the sex more likely to emigrate also suffering higher rates of tuberculosis mortality (Hinde, 2015). Alice Reid and Eilidh Garrett demonstrated such a phenomenon on the Isle of Skye in the late nineteenth century, where males showed a greater tendency to emigrate and also suffered higher tuberculosis mortality (Reid and Garrett, 2018).

Whether similar forces were at work in earlier periods remains unclear. Before the mid-nineteenth century the main risks to urban migrants may have been acute diseases that were lethal enough to overwhelm the defences even of very robust and well-fed young adults. These included plague (before the 1670s), smallpox (before c.1800), typhus and typhoid, diseases against which the main defence was prior exposure. These diseases were also acute, and therefore offered little scope for return migration. Infected individuals were quickly rendered too ill to travel, or were barred from most transport options because of fears of contagion. In contrast, tuberculosis sufferers had time to make decisions about whether to migrate to a town, and when to return home.

These differing disease patterns and migrant responses may have had large effects on death and burial patterns in urban centres, because young adult migrants comprised a high proportion of urban populations (Table 3). Where acute and very virulent diseases predominated then we might expect to find a preponderance of otherwise healthy and well-built young adult migrants amongst adult burials in seventeenth and eighteenth-century cities. However as these more lethal diseases receded across the nineteenth century (for reasons that remain poorly understood but which include vaccination and other public health measures, and possibly improvements in personal hygiene), then healthy migrant and salmon bias effects may have played a larger role. In this case then we would expect young adult migrants *living in towns* to have comprised a group that was doubly selected for robust health, and that displayed very low death rates in early adulthood compared with their urban-born contemporaries. In this scenario some of the health risks associated with migration to towns would be masked by return urban-rural migration, and would be under-estimated in studies of urban death rates or burials. Moreover, such processes would have operated to reduce urban-rural gradients in observed adult mortality between towns and their migrant hinterlands (Farr, 1885; Hinde, 2015). Importantly, urban-rural migration could also distort rural patterns where urban-born children and adolescents were sent to rural locations for nursing (Clark, 1987; Levene, 2007) or to labour in remote mills (Gowland et al., 2018).

## 6 Conclusions

The core finding of this article is that industrialisation was not associated with a pronounced worsening of mortality levels in English cities. The *proportion* of the population exposed to urban conditions certainly rose over the period c.1760-1900. Communities that shifted from rural activities to industrial ones also experienced often marked deteriorations in death rates (Huck, 1995; Wrigley et al., 1997, pp. 273-5). However for urban populations as a whole, survival prospects actually improved during the Industrial Revolution, compared with previous centuries. Improvements were most marked for those groups who were most susceptible to urban diseases-infants, young children and rural-urban migrants. There was a partial reversal of these improvements for young children (ages 1-4 years) in the decades of the 1830s – 1860s, however this reversal was not confined to urban or industrial centres.

This article has not attempted to explain most of the causes of these mortality trends, which were multi-factorial and remain poorly understood. Nor have I touched at all on the roles of medical theories and public health movements. Instead I have drawn attention to the roles of several key factors that were broadly independent of income and physical environment: breastfeeding practices; specific measures to control smallpox; and changes in the characteristics of smallpox (in the seventeenth and eighteenth centuries) and scarlet fever (in the period c.1830 – 70). Neither smallpox nor scarlet fever mortality was closely associated with nutritional status, sanitation or poverty. Instead it appears that novel genetic variants arose, with higher virulence and/or infectiousness.

The second main ambition of the article was to flag some of the challenges in studying levels and changes in mortality during the Industrial Revolution, and to suggest areas where interdisciplinary collaboration may be especially fruitful. Both demographers and archaeologists rely primarily on evidence of urban burials, and these can be misleading without considerable contextualisation. Urban populations were characterised by unusual age structures and by unusual patterns of age-specific mortality, including very high levels of mortality in early childhood and possibly in early adulthood relative to older ages. Age structures cannot be reliably reconstructed from burial patterns, and mortality rates cannot be estimated without census data or detailed reconstruction of local communities. The complexities of rural-urban migration flows and intra-urban residential mobility (including extra-parochial burials) had potentially strong effects on the composition of local burials and could introduce strong socioeconomic biases. In addition, the evidence of very marked falls in infant and child mortality between the eighteenth and nineteenth centuries complicates the interpretation of skeletal evidence drawn from burial grounds that were in use over long periods, unless there is additional evidence with which to date individual burials (DeWitte and Stojanowski, 2015; Ives and Humphrey, 2017; Nitsch et al., 2011).

For demographic historians the main contributions of archaeology probably lie in the capacity to *explain* urban mortality patterns. I have argued that migration, breastfeeding patterns and pathogen evolution were key influences on urban mortality levels and trends during industrialisation. However historical evidence for breastfeeding patterns remains largely indirect (inferred from anecdotal evidence, burials of nurse children, and birth intervals, before the twentieth century). The roles of genetic change in pathogens also remain largely conjectural. These are areas where historians can make only limited contributions, and where archaeological techniques are crucial. Osteological and dental remains can provide direct evidence of weaning practices, and of the genomic identity of ancient pathogens (e.g. Henderson et al., 2014; Newman and Gowland, 2017; Nitsch et al., 2011) . They are also invaluable in distinguishing migrants from urban-born individuals, as well as providing evidence of health status not captured by mortality rates (e.g. Gowland et al., 2018; Lewis, 2002; Mays et al., 2009, 2008; O’Donoghue et al., this volume; Pinhasi et al., 2006). The historical demography of urban populations poses enormous methodological challenges, that can only be addressed fully by inventive approaches that triangulate evidence gleaned from an array of sources, and that are acutely sensitive to the problems of interpretation posed by high volumes of migration and residential mobility, and complex childcare arrangements and burial practices. Closer collaboration between historians and archaeologists is essential to identify key tractable questions, and to exploit all of the available evidence.

## Figures and Tables

**Table 1:** Life expectancy in English cities with populations of 100,000 or more^a^

City	1838-44	1851-60	1861-70	1871-80	1881-90	1891-1900
London	36.7	38.0	37.7	40.4	42.6	43.7
**Liverpool**	28.3	30.3	28.7	33.4	35.4	37.5
**Manchester**	29.8	32.2	32.3	34.9	37.3	38.3
**Birmingham**	35.5	37.1	37.5	39.0	42.0	41.9
Bristol	35.8	39.1	39.4	41.5	45.7	48.1
**Leeds**	35.1	35.3	34.6	37.3	40.3	41.7
**Sheffield**	36.1	35.2	35.5	37.3	40.2	41.6
**Bradford**		35.3	36.5	38.2	41.9	39.5
**Newcastle**			34.1	36.8	40.3	41.5
**Salford**			34.9	34.6	36.6	36.5
**Hull**				40.3	42.6	44.0
Portsmouth				43.5	45.3	45.7
**Blackburn**					39.2	41.1
**Bolton**					41.4	41.9
Brighton					45.6	46.4
**Leicester**					42.6	44.0
**Nottingham**					41.8	44.1
**Oldham**					38.6	39.5
**Sunderland**					40.7	41.1
Cardiff						45.2
Norwich						46.4
**Preston**						38.6
England and Wales	40.4	41.1	41.2	43.0	45.3	46.1

a Towns in northern and midland counties appear in bold. Estimates refer to municipal boroughs or urban sanitary districts and are calculated as population-weighted averages of life expectancies in registration districts (RDs) associated with each city. Cities were included in the sample when the population of the administrative city reached 100,000 in the census year that opened the decade. Estimates of e_0_ for England and Wales and London in the period 1838-44 refer to Woods’ figures for the decade 1841-50 (Woods, 2000). Sources: *8th and 9th Annual Reports of the Registrar-General* (P.P. 1847/8, XXV) (values for English cities 1838-44); Woods, 1997 (1851-1900); Szreter and Mooney, 1998 Table 5 (values for Glasgow); Woods, 2000, Table 9.3 (England and Wales and London).

**Table 2:** Infant mortality rates in European cities c. 1865.

Unit	Deaths/100 births^a^	Period	Source
England and Wales	15.2	1861-70	Human Mortality Database
London	16.0	1861-70	Woods, 1997
Liverpool	21.0	1861-70	This paper
Manchester	18.8	1861-70	This paper
Birmingham	17.1	1861-70	This paper
Bristol	15.7	1861-70	This paper
Denmark	13.4	1861-70	Williams and Mooney, 1994
Copenhagen	20.9	1861-70	Williams and Mooney, 1994
provincial Danish towns	15.1	1861-70	Williams and Mooney, 1994
France	18.8	1860-64	Human Mortality Database
Paris	19.0	1861-65	Preston and van de Walle, 1978
Lyon	17.2	1861-65	Preston and van de Walle, 1978
Marseilles	20.7	1861-65	Preston and van de Walle, 1978
Germany	23.3	1861-70	Gehrmann, 2011
Munich	40.0	1862-69	Brown and Guinnane, 2018
Berlin	30.4	1875-80	Gehrmann, 2011
Frankfurt	22.0	1875-80	Gehrmann, 2011
Hamburg	21.9	1875-80	Gehrmann, 2011
Netherlands	19.7	1875-79	Wolleswinkel-van den Bosch et al., 2000
Amsterdam	21.6	1875-79	Wolleswinkel-van den Bosch et al., 2000
Rotterdam	23.2	1875-79	Wolleswinkel-van den Bosch et al., 2000
Norway	11.0	1860-64	Human Mortality Database
Kristiana (Oslo)	17.0	1861-65	Hubbard, 2000
Bergen	16.2	1861-65	Hubbard, 2000
Spain	18.4	1860-62	Ramiro-Fariñas and Sanz-Gimeno, 2000
Madrid	26.2	1860-62	Ramiro-Fariñas and Sanz-Gimeno, 2000
Sweden	13.6	1861-70	Woods, 1993
Stockholm	26.8	1861-70	Woods, 1993

a Values refer to a range of spatial units that sometimes included substantial rural elements: see sources for details.

**Table 3:** Migrants as a percentage of the adult population of London, 1565 – 1851, and other large English cites in 1851.

City	Period	% born outside London	Sample	Source
London	1565-1644	78 %	Adult witnesses, London Consistory Court	Elliot, 1978
London	1580-1639	74 %	Adult male witnesses Stepney and Whitechapel church courts	Cressy, 1970
London	1601-1690	80 %	Male apprentices	Wareing, 1980
London	1665-1725	69 %	Adult female witnesses, London church courts	(Earle, 1989)
London	1683-1759	62 %	Indentured servants (male and female)	Wareing, 1981
London	1774-1781	75 %	Married men and women, Westminster General Dispensary	George, 1984, p.118
London	1773-1775	39 %	Indentured servants (male and female)	Wareing, 1981
London	1851	54 %	Adults aged 20 and over	1851 census^a^
Liverpool	1851	77 %	Adults aged 20 and over	1851 census^a^
Manchester with Salford	1851	72 %	Adults aged 20 and over	1851 census^a^
Birmingham	1851	56 %	Adults aged 20 and over	1851 census^a^
Bristol	1851	65 %	Adults aged 20 and over	1851 census^a^
Leeds	1851	42 %	Adults aged 20 and over	1851 census^a^
Sheffield	1851	49 %	Adults aged 20 and over	1851 census^a^

a *Census of Great Britain, 1851, Population Tables, II* (Vol. I. BPP 1852-53 LXXXVIII Pt [1691.I]), p. clxxxiii.
